# Association of Vital Exhaustion with Risk Factors for Cardiovascular Diseases, Quality of Life and Lifestyle in 41–44-Year-Old Muscovite Men

**DOI:** 10.3390/ijerph18189691

**Published:** 2021-09-14

**Authors:** Marina B. Kotova, Vyacheslav B. Rozanov, Anton R. Kiselev, Sergey A. Maksimov, Oxana M. Drapkina

**Affiliations:** National Medical Research Center for Therapy and Preventive Medicine, 101990 Moscow, Russia; vbrozanov@gmail.com (V.B.R.); antonkis@list.ru (A.R.K.); SMaksimov@gnicpm.ru (S.A.M.); drapkina@bk.ru (O.M.D.)

**Keywords:** vital exhaustion, psychosocial stress, cardiovascular diseases, hypertension, physical activity, alcohol, hand-grip dynamometry, lifestyle, quality of life

## Abstract

(1) Background: Vital exhaustion (VE) is no less of an important risk factor (RF) for cardiovascular diseases (CVD) and cardiovascular events than the well-known RFs. Insufficient knowledge of the relationship between VE and CVD RF, quality of life, and lifestyle was the rationale for this study. (2) Methods: We examined 301 Muscovite men 41–44 years of age. The categorization of RFs for CVD was carried out in accordance with conventionally considered criteria. In order to evaluate the lifestyle and quality of life in study participants, we were offering them a self-filling questionnaire developed by I.A. Gundarov. The presence of VE signs was assessed using a 14-item short version of the Maastricht Vital Exhaustion Questionnaire scale (MVEQ). All study subjects were classified into three ordered groups depending on the distribution of VE indicators by tertiles: Group 1 consisted of men with a low VE (0–2 points), Group 2 included males with a medium VE score (3–5 points), and Group 3 comprised subjects with high VE scores (6–14 points). To analyze the obtained data, we used one-way analysis of variance (ANOVA), Pearson’s chi-squaredtest (χ^2^), Goodman and Kruskal’s gamma, and linear regression analysis. (3) Results: We established that every third male (36.8%) had VE signs, while 10.6% of men had high VE levels. With an increase of VE in men, the frequency of arterial hypertension (AH) was increasing as well, and it was significantly higher in men with a high VE compared to their peers with a low VE (48.4% versus 33%; *p* = 0.03). A significant linear relationship was discovered between VE levels and excessive alcohol consumption (*p* = 0.001). The strongest linear associations were found between the VE level, and both psychosocial stress indicator and the amount of consumed ethanol. Self-assessment of personal happiness, job and sleep satisfaction, residential living conditions, and spiritual needs, as well as psychosocial stress indicator, total amount of consumed ethanol, and muscle strength (hand-grip dynamometry), were independent determinants of the VE level, and, collectively, they explained 46.6% of its variability. The greatest contribution to VE was made by the personal happiness level, explaining 25.5% of its variability. The proportions of the VE variance uniquely explained by various factors were as follows: 9.3% by the psychosocial stress, 4.9% by job satisfaction, 2.8% by sleep satisfaction, 2.3% by total consumption of ethanol, 1.6% by muscle strength, 1.1% by living conditions in the residential neighborhood, and just 0.8% by spiritual needs. (4) Conclusion: High VE levels in 41–44-year-old men are associated with AH, sedentary behavior, excessive alcohol consumption, and lower values of most indicators of both lifestyle and quality of life.

## 1. Introduction

Psychosocial risk factors (RFs), along with conventional RFs, increase the risk of developing cardiovascular diseases (CVD), impair CVD prognosis, and prevent strong adherence to treatment and efforts to improve lifestyle, both in patients and in the population as a whole [1]. According to the European Guidelines on Cardiovascular Disease Prevention in Clinical Practice [1], psychological factors include parameters caused primarily by stressful impacts: anxiety, depression, hostility, vital exhaustion (VE), and sleep disorders [1]. 

In their meta-analysis, Cohen et al. (2017) showed that VE is a RF for CVD that is no less important than other conventionally accepted factors [2].

The term ‘*vital exhaustion*’was first introduced in 1987 by Appels et al. in a prospective Rotterdam Civil Servants Study. VE was defined as feeling excessively tired, exhausted, irritable, and demoralized [3]. Over the past decade, VE has attracted considerable attention from researchers due to its adverse effects on health and well-being [4,5,6]. In particular, the conducted studies have shown that VE was an independent RF for the development and progression of CVD [2,4,5,6,7], and men were at greater risk of developing CVD than women [8,9]. At present, VE is an understudied CVD RF. It is an early indication of acute myocardial infarction and an RF for coronary heart disease and cerebrovascular events [2,6,10,11,12]. The cause of VE is not fully understood as yet. It is assumed that VE develops as a result of a breakdown in adaptation to chronic stress [8,13]. People experiencing periodic or prolonged stress are more likely to exhibit unhealthy behavior, such as undereating or overeating, low physical activity, and alcohol abuse, which, in turn, negatively affects their health [14]. Some studies suggested that poor health was associated with social and occupational stress, low social support, and VE [15]. 

Consequently, the adverse impact of VE on human health and well-being, as well as its relationship with CVD, can be mediated by changes in lifestyle and deterioration of the quality of life.

Hence, currently, there is a dire need for studying VE in human populations and its impact on CVD morbidity and prognosis. In particular, insufficient knowledge of the relationship between VE and CVD RFs, quality of life, and lifestyle served was the rationale for this study. 

The objective of our research was to evaluate the associations of VE with CVD RFs, lifestyle, and quality of life in 41–44-year-old Muscovite men.

## 2. Materials and Methods

### 2.1. Study Participants

In 1983, a sample was formed for a long-term prospective observational study on the dynamics of main CVD RFs. The sample included fifth grade school students residing in Moscow. At baseline, 23 out of 79 schools were randomly selected. The chosen schools enrolled 1182 boys, 11–12 years of age, attending the fifth grade. A total of 1005 individuals were examined, which constituted 85% of the selected population. Their average age was 11.9 ± 0.11 years. Over 32 years of prospective study, 7 medical examinations were carried out at different intervals. Our study participants were represented by men selected for subsequent analysis at their seventh visit to a physician within the framework of the above-mentioned prospective study.

### 2.2. Measurement Methods

The procedure of medical examination encompassed the following components: a standard questionnaire with passport information, educational status, social status, personal anamnesis, family medical history, lifestyle (physical activity, cigarette smoking, and alcohol consumption); threefold measurement of blood pressure (BP); measurements of body mass (BM) and height, as well as of waist and hip circumference (WC, HC); and determination of the levels of total cholesterol, high density lipoprotein cholesterol (HDL cholesterol), triglycerides, and low density lipoprotein cholesterol (LDL cholesterol). The body mass index (BMI) was used to evaluate body mass excess (BME) and obesity, and the ratio of WC to HC was calculated to characterize an abdominal obesity.

The arterial hypertension (AH) group included men with a BP ≥ 140/90 mmHg, as well as with a BP < 140/90 mmHg but receiving antihypertensive treatment. BME was characterized by BMI 25 ≥ kg/m^2^, whereas obesity was categorized at BMI ≥ 30 kg/m^2^. The group of abdominal obesity included subjects with WC ≥ 94 cm. The risk categories for the blood lipid panel were formed on the basis of borderline high values *sensu*, the classification presented in the third report of the expert group of the National Cholesterol Education Program (NCEP) on detection, assessment, and therapy of high cholesterol levels in adults—Adult Treatment Panel III (ATP III) [16]. 

The presence of VE signs was established via a 14-item short version of the Maastricht Vital Exhaustion Questionnaire scale (MVEQ) [3]. The Maastricht Questionnaire includes 14 items that describe various aspects of VE with a possible score range from 0 to 14 points. In order to ensure comparability and sufficient sample sizes in the compared groups, all study participants were classified into three ordered groups depending on the distribution of VE indicators by tertiles. Group 1 consisted of men with a low VE (0–2 points), Group 2 included males with a medium VE score (3–5 points), and Group 3 comprised the subjects with high VE values (6–14 points).

The Reeder Stress Inventory (L.G. Reeder et al. 1969), adapted by O.S. Kopina et al. (1989), was used as the method of express diagnosing of the psychosocial stress (PS) level [17]. 

To evaluate the lifestyle and quality of life in the study participants, we were offering study subjects a self-filling questionnaire developed by I.A. Gundarov [18]. The following parameters were analyzed: material well-being in a household; personal earnings; living conditions of a subject; environmental conditions in the area of a subject’s residence; family and children; variety of a consumer food basket and satisfaction with food; love and sexual feelings; spiritual needs, social support and communication with friends; and the type of work and position in society. The quality-of-life indicators were assessed by the respondents on a 100-point scale.

In addition to the I.A. Gundarov’s questionnaire, such behavioral habits as smoking, alcohol consumption, and physical activitylevelwere assessed to identify the lifestyle of each study participant.

To measure physical activity, the International Physical Activity Questionnaires (IPAQ) were employed [19]. Physical activity categories were formed in accordance with IPAQ recommendations [20]. Men smoking at least one cigarette a day were considered regular smokers. To assess alcohol consumption, we used standard questionnaires borrowed from the *Russian Longitudinal Monitoring Survey* (RLMS) [21] and the *Survey on Stress, Aging and Health in Russia* (SAHR) [22]. Questions included the following items: frequency and volume of alcohol consumption (per day, per week, per month) throughout the year, and categorization of alcoholic beverages (beer, wine, spirits, etc.). Alcohol consumption has been converted to pure ethanol, and excessive alcohol use was stated for those who consumed over 168 g of pure ethanol per week.

### 2.3. Statistical Analyses

Descriptive statistics presented in tables and a figure have the following notations: n is the sample size (number of subjects) in a group; % represents the proportion of subjects of their total number in a group; M is an arithmetic mean; and 95% CI denotes a 95% confidence interval. To check quantitative variables for normality of distribution, we used descriptive statistics, histograms of residuals, and normal probability plots (Q-Q plots). The homogeneity of variances was checked using the Levene’s test. One-way analysis of variance (ANOVA) was used to test for a linear association among the ordered categories of the factor variable and dependent continuous variables. The Holm–Bonferroni correction was applied to eliminate a power drop with a large number of dependent variables. Group comparisons, corrected for differences in one or more variables, were performed using the least squares method in the SAS PROC GLM procedure. The hypothesis on dependence of the distribution of subjects with CVD risk factors on the magnitude of VE was tested using the Pearson’s chi-squared test (χ2), followed by pairwise comparisons of proportions via Z-test. The strength and direction of the relationship between ordinal and dichotomous variables was evaluated by means of Goodman and Kruskal’s gamma test (gamma coefficient). The association of VE with the studied parameters was assessed using multiple linear regression with stepwise input of independent variables into the model. The variance inflation factor (VIF) was used to check for collinearity.

The critical level of statistical significance (*p*) in our study was 0.05. Statistical data processing was performed using SAS (Statistical Analysis Software) 9.0 and IBM SPSS Statistics version 23 software for Windows.

## 3. Results

After 32 years, out of 1005 males, just 301 (30%) representatives of the original population sample were examined on their seventh medical examination visit. The average age of surveyed men at the moment was 42.9 years.

**Figure 1:**
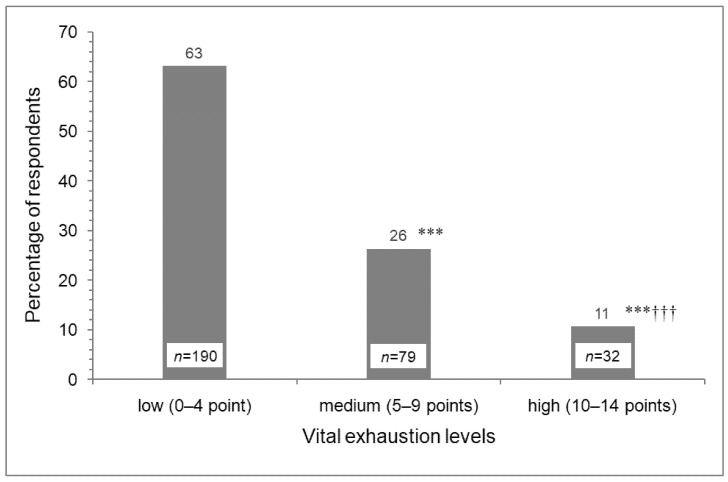
Distribution of male respondents among the vital exhaustion levels (1—low, 2—medium, 3—high) (*N* = 301). Unambiguous criteria were chosen to categorize the vital exhaustion. *** *p* < 0.001 versus level 1; ††† *p* < 0.001 versus level 2. Paired comparisons are made using Z-criteria with Holm-Bonferroni correction.

We estimated the frequency of different VE parameters in the surveyed sample of 41–44-year-old men using unambiguous criteria [3]. In particular, 26.2% of surveyed men were characterized by a medium VE level, whereas 10.6% had high VE scores (Figure 1).

The frequency distribution of CVD RFs, according to ordered VE levels, is presented in Table 1. This table suggests the relationship between AH and VE level (Pearson’s χ^2^ = 6.043, *p* = 0.049) and association among excessive alcohol use and VE level (Pearson’s χ^2^ = 12.155, *p* = 0.002). These dependences are linear (χ^2^ for trend = 4.693, *p* = 0.030; χ^2^ for trend = 11.946, *p* = 0.001, respectively), i.e., with increase in VE level, there is a rise in both AH frequency and frequency of excessive alcohol consumption in 41–44-year-old men. AH incidence was statistically significantly higher among men with a high VE compared with low and medium VE levels, and the frequency of excessive alcohol use was higher among men with medium and high VE levels versus low VE level. The established a directly proportional relationship between VE level and indicated RFs wereweak for AH (gamma = 0.193; *p* = 0.045) and moderately strong for excessive alcohol use (gamma = 0.385; *p* < 0.001).

Comparative analysis of the studied CVD risk markers in groups of men with different VE levels (Table 2) demonstrated statistically significant unidirectional linear trends of VE versus group average BMI, hand-grip dynamometry, HDL cholesterol, volume of consumed alcohol, duration of sedentary behavior, and PS level. The strongest linear relationship was established between VE level and PS values.

Previously identified intergroup differences in BMI and HDL cholesterol (Table 3) disappeared after adjusting the CVD RFs in terms of PS level. 

The comparative analysis of lifestyle and quality of life indicators in groups of men with different VE levels (Table 4) exhibited the existence of statistically highly significant inverse linear relationships between the group means for most of the studied variables and ordered VE levels: i.e., with an increase in VE level, the values of the studied indicators were declining. The greatest decrease in the studied parameters was observed in the group of men with a high VE level. 

Taking into account the close relationship between VE and PS (Table 2), we corrected intergroup differences in quality of life and lifestyle indicators for the PS level, which did not significantly affect previously identified (Table 4) inverse linear trends and intergroup differences for most of the studied indicators (Table 5). The exceptions were the lack of stress in the workplace, satisfaction with the authorities, personal safety, and satisfaction with housing conditions, the VE effect that has reduced to statistically insignificant values (Table 5).

Multiple linear regression was used to assess the relationship between VE and the studied parameters. Initially, 27 independent variables, characterizing the lifestyle and quality of life, and statistically significantly correlating with VE, were introduced into the regression model. In the final model, as a result of stepwise selection, just eight independent variables remained (Table 6). We evaluated the regression model as consistent and explanatory, since the number of used observations was much greater than the number of independent variables in the model. The multiple correlation coefficient was statistically significant (R = 0.695; *p* = 0.049), the regression coefficients were also significant, and VIF values implied the absence of multicollinearity (Table 6). Negative values of regression coefficients (B and beta) for PS implied a directly proportional relationship with VE caused by inversely proportional values of the scales for PS and VE. The VE level was directly proportional to the PS level and inversely proportional to other explanatory variables, with the exception of the amount of consumed alcohol (ethanol). The greatest contribution to VE was made by the personal happiness level, explaining 25.5% of its variability. The proportion of the VE variance uniquely attributable to PS was just 9.3%. Further, in decreasing order of the explained variance proportion are: the employment satisfaction, sleep satisfaction, total amount of consumed ethanol, muscle strength (hand-grip dynamometry), living conditions in the residential neighborhood, and spiritual needs. Altogether, explanatory variables accounted for 46.6% of the variability in VE levels. 

## 4. Discussion

Our study subjects were represented by men selected for subsequent analysis at their seventh visit to a physician within the framework of the 32-year prospective study.

The results of our research showed that the VE level in 41–44-year-old men was associated with excessive alcohol consumption and increased likelihood of developing AH.

Additionally, the VE level in 41–44-year-old men, regardless of their PS score, was associated with an increase in the amount of consumed alcohol and duration of sedentary behavior, along with reduction in their physical activity and muscle strength. The linear association of VE with BMI and HDL cholesterol was caused by the confounding effect of the PS.

An increase in the VE level in 41–44-year-old men was associated with a decrease in most indicators of quality of life and lifestyle after their adjustment for the PS score. The linear relationships between VE and lack of stress in the workplace, satisfaction with the authorities, personal safety, and satisfaction with housing conditions were also caused by the confounding effect of the PS. 

The most significant determinants of the VE in 41–44-year-old men were personal happiness level, PS score, satisfaction with employment and sleep, total amount of consumed ethanol, muscle strength, living conditions in the residential neighborhood, and spiritual needs.

The results of our study suggested that over 25% of surveyed men had a medium VE level, while every tenth study subject had a critically high VE value. Our findings partially agree with the results of the study conducted in Siberia on Tyumen men in terms of the critical VE level (12.3%), but are lower than the fraction of Tyumen males with a medium VE level (38.6%) [23], and are even lower than the level of corresponding indicators revealed by some studies conducted in Europe, where the proportion of men with a medium VE level was 51.7% and with a critical high VE level was 31.5% [24]. The lower prevalence of VE in the Moscow area, as compared to the Tyumen region, may have been caused by the higher quality of life in Muscovites and more severe climatic conditions in the Siberian region. We hypothesize that a higher level of VE in the male population of the Netherlands, compared with Moscow men, could be influenced by a more intense workload associated with their professional activities, an insufficient social circle, and also, possibly, by the nonexistent or weak social support from relatives and friends. 

### 4.1. Vital Exhaustion versus Cardiovascular Risk Factors

The direct dependence of AH frequency on VE level that we have established in men was consistent with the results of numerous foreign and domestic studies, which drew attention to the significant contribution of VE to high systolic blood pressure, coronary artery disease, and development of cardiovascular events [2,6,7,10,11,25,26]. Van Diest et al. and Balog et al. proposed that VE could represent a more somatic dimension of distress [4,27]. According to the Hungarian study, VE was the only independent, significant, and reliable psychological predictor of the recurrence of vascular events [26]. 

The lack of relationship between the VE level and BP in our study was apparently caused by the fact that the majority of males with AH (71.1%) received antihypertensive medicamentous therapy, which was reflected in their average systolic and diastolic BP measurements in all groups.

The relationship among the VE level and excessive alcohol consumption revealed in our study was consistent with other studies that established direct relationships between the VE and total amount of consumed ethanol, sedentary lifestyle, and smoking [2,28,29]. These dependences seem very predictable, since men with high VE are more likely to offset the frustration and demoralization, inherent in VE, by choosing the fastest and seemingly most affordable way to correct this situation by means of the alcohol abuse. Excessive fatigue and weakness, subjectively experienced by men, were also represented in our study by entirely objective characteristics: a reduction in hand-grip dynamometry and an increase in sedentary behavior with higher VE levels. However, despite multiple studies showing an increase in the frequency of smoking with an increase in the VE level in various population groups [2,28,29], we did not encounter such relationship. Perhaps, this was due to insufficient number of observations.

The 2008 study by Bryant et al. [30] and 2011 study by Igna et al. [28] revealed the direct dependence of BMI on the VE level, which was not supported by the 2012 study of Iversen et al. [31]. Our research demonstrated an inverse relationship of the kind: specifically, higher VE corresponded to lower BMI values. Enlarged HDL cholesterol content with an increase of VE, also demonstrated in our research, was not consistent with the results of the study by Koertge et al. [32], in which high VE was associated with an enlarged level of HDL cholesterol. Hence, we conclude that the established association of VE with BMI and HDL cholesterol was caused by the confounding effect of PS. 

We think that the strongest established relationship between the levels of VE and PS was quite expected; this finding has supported the 2008 study by Bellingrath et al., who demonstrated that VE could be viewed as a consequence of prolonged stress and lack of resources required to adapt to the stress [33]. Men with increased VE were spending shorter time on hobbies and spiritual needs, which has indicated a serious deterioration in their quality of life and impoverishment of their lifestyle. The latter was expressed in demoralization, loss of strength, and excessive fatigue. Hobbies and spiritual needs of a person are protective resources or coping strategies aimed, among other things, at overcoming psychoemotional stress [34].

### 4.2. Vital Exhaustion versus Sociopsychological Environment, Lifestyle and Quality of Life

Since VE is an indicator of both physical and psychological well-being, a noticeable decline in the quality of life and lifestyle impoverishment in men with increases in VE can be traced for nearly all studied indicators. It is important to note that correction for the PS score did not affect previously identified trends and intergroup differences for most of the studied indicators, with the exception of the following: lack of stress in the workplace, satisfaction with the authorities, personal safety, and satisfaction with housing conditions. This finding implies that the primary reason for the relationship between a high level of VE and these indicators is stress per se, caused by dissatisfaction with individual (work, housing) and social (satisfaction with the authorities, personal safety) living conditions [35] over a long period of time, which leads to physical and emotional exhaustion, once again confirming an assumption by S. Bellingrath that VE is a consequence of prolonged stress [8,33] associated in men with existing living conditions. 

It should be pointed out that an aggravation of VE in men has a negative impact on the most important parts of their lives. Fatigue and loss of energy, which characterize VE, significantly affect one of the main priorities of their lives: work and professional development, which is expressed in a reduction of job satisfaction and deterioration in the type and conditions of employment. Besides, psychosocial risks associated with work are among the most challenging issues of occupational health and work safety that can significantly affect the physical and mental health of employees and have a negative impact on the performance of organizations (absenteeism, high turnover, low labor productivity) and even the entire national economy [36,37]. Interpersonal relationship issues (with family, colleagues and friends) are also aggravated with an increase in VE.

It should be emphasized that an increase in VE in men, observed in our study, was associated with changes in their eating habits, which most likely related to a somewhat significant association between VE and stress, since activation of the adrenal sympathetic cerebral systems during stress leads to the release of adrenaline and norepinephrine, which in turn may suppress appetite [38]. Hence, we could assume that appetite reduction was associated with a decrease in dietary diversity, availability, and satisfaction with the amount of consumed food. Consequently, all of these indicators, revealed with an increase in VE (Table 5), could significantly affect the eating habits, most often, impoverishing their quality and reducing dietary diversity, based on self-evaluations by the respondents. A reduction in BMI and an increase in HDL cholesterol in men with a pronounced VE, prior to the correction for the PS level, implies a confounding effect of the stress factor on the development of VE. 

An increase of VE in men was associated with a decline in satisfaction experienced from the support by significant social environment (family, relatives, friends), as well as from communication with friends and position in the society. The physical and emotional conditions in men, caused by VE, considerably limited their social contacts and reduced the level of their communication, thus significantly impoverishing their quality of life. Social support from the family and friends is a strong and persistent determinant of mental health in adults, and this association has been noted in many studies [8,39]; on the contrary, social isolation may contribute to the onset of heart failure, which was shown on the general population of the United States via an increase in VE [8].

We demonstrated that PS, job satisfaction, sleep satisfaction, total volume of consumed ethanol, muscle strength, living conditions in the residential neighborhood, and spiritual needs were significant determinants of VE, albeit to a lesser extent than the personal happiness. In contrast, in the Men Stress 40+ study [8], chronic stress was the only significant VE predictor.

## 5. Conclusions

The results of our study assessing the association of vital exhaustion with lifestyle and quality of life, as well as with risk factors for cardiovascular diseases in 41–44-year-old Muscovite men, disclosed that high level of vital exhaustion in these men was associated with such cardiovascular disease risk factors as arterial hypertension, sedentary behavior, and excessive alcohol consumption and lower values of most indicators of lifestyle and quality of life. 

The most significant determinants of the vital exhaustion in 41–44-year-old men were personal happiness, psychosocial stress, job satisfaction, sleep satisfaction, total volume of consumed ethanol per unit time, muscle strength, living conditions in a residential neighborhood, and spiritual needs. All of these need to be taken into account, along with other behavioral and psychosocial risk factors for cardiovascular diseases, when developing the programs aimed at strengthening and maintaining the population health. An important implication of our research is that additional studies are needed to extend our findings to women, and men of other ages and from other regions of Russia.

### Study Strengths and Limitations

Our research was carried out on a homogeneous group (in terms of age and gender) living in similar conditions. In Russia, there have been no similar studies examining the associations of vital exhaustion with risk factors for cardiovascular diseases, quality of life and lifestyle. In the available worldwide scientific publications, such studies are also extremely rare and are mainly devoted to individual aspects of the problem under study.

However, this study is not without its drawbacks. A significant limitation of the study is the low response rate of the participants, compared with the original study. The reasons for this fact could be significant socioeconomic and political changes in the country that have occurred over 32 years. As a result, manystudy subjects surveyed in 1984 (as 11-year-old school students) changed their place of residence; hence, their contact information was no more available. Besides, high male mortality (for various reasons) in the 1990s in Russia was another negative factor. Unfortunately, it is not possible to estimate how low response rate could have influenced our results due to the lack of any regularities in the ‘*contact information loss*’. However, we do not repudiate that the low response rate could have affected the obtained results. In fact, the conducted study was cross-sectional in its design, which did not allow evaluating the results in terms of cause-and-effect relationships. In addition, it should be noted that some of the parameters under study were quantitively assessed using the questionnaires—i.e., their values could only be true given the assumption of honest responses.

Additionally, one of the limitations of our study is the fact that some of the questionnaires we used were not validated.

## Figures and Tables

**Table 1:** Distribution of risk factors in groups of males with different vital exhaustion levels.

Risk Factors		Vital Exhaustion Levels			Chi-Squared Tests	
		1 (*n* = 97)	2 (*n* = 109)	3 (*n* = 93)	Linear-by-Linear Association	Pearson’s Chi-Square
Overweight status + obesity	yes	71 (73)	73 (67)	57 (61)	x^2^ = 3.05 *p* = 0.081	x^2^ = 3.06 *p* = 0.26
	no	26 (27)	36 (33)	36 (39)		
Abdominal obesity	yes	50 (52)	54 (50)	40 (43)	x^2^ = 1.37 *p* = 0.242	x^2^ = 1.52 *p* = 0.468
	no	47 (48)	55 (50)	53 (57)		
Arterial hypertension	yes	32 (33)	37 (34)	45 (48) *†	x^2^ = 4.69 *p* = 0.030	x^2^ = 6.04 *p* = 0.049
	no	65 (67)	72 (66)	48 (52)		
Dyslipidemia	yes	73 (75)	83 (76)	65 (70)	x^2^ = 0.69 *p* = 0.405	x^2^ = 1.15 *p* = 0.562
	no	24 (25)	26 (24)	28 (30)		
Current smokers	yes	40 (41)	50 (46)	44 (47)	x^2^ = 0.71 *p* = 0.399	x^2^ = 0.79 *p* = 0.675
	no	57 (59)	59 (54)	49 (53)		
Low physical activity	yes	27 (28)	24 (22)	27 (29)	x^2^ = 0.03 *p* = 0.863	x^2^ = 1.51 *p* = 0.471
	no	70 (72)	85 (78)	66 (71)		
Alcohol consumption	yes	76 (78)	86 (79)	80 (86)	x^2^ = 1.78 *p* = 0.182	x^2^ = 2.27 *p* = 0.321
	no	21 (22)	23 (21)	13 (14)		
Excessive alcohol consumption	yes	11 (11)	26 (24) *	30 (32) ***	x^2^ = 11.95 *p* = 0.001	x^2^ = 12.16 *p* = 0.002
	no	86 (89)	83 (76)	63 (68)		

Data are presented as n (%).Hereinafter, the 3 groups are ordered according to the increasing level of vital exhaustion (VE): 1—low VE level; 2—medium VE level; 3—high VE level. Data are presented as n (%). * *p* ˂ 0.05, *** *p* ˂ 0.001 (versus level 1); † *p* ˂ 0.05 (versus level 2). Paired comparisons are made using Z-criteria with Holm–Bonferroni correction.

**Table 2:** Comparative analysis of CVD risk markers in groups of males with different vital exhaustion levels.

Studied Indicators	Vital Exhaustion Levels			F-Test for Trend
	1 (*n* = 97)	2 (*n* = 109)	3 (*n* = 93)	
Age, years	42.9 (42.8–43.0)	43.0 (42.9–43.1)	42.9 (42.8–43.0)	F = 0.60, *p* = 0.440
SBP, mmHg	121 (118–123)	122 (119–124)	125 (121–128)	F = 3.17, *p* = 0.076
DBP, mmHg	82 (80–84)	82 (80–84)	84 (81–86)	F = 1.67, *p* = 0.198
Pulse, beats per minute	73 (71–75)	75 (73–77)	75 (72–77)	F = 0.96, *p* = 0.328
BMI, kg/m^2^	28.7 (27.6–29.7)	27.5 (26.6–28.4)	26.9 (25.9–27.8) *	F = 6.85, *p* = 0.009
WC, cm	96.3 (93.5–99.1)	93.8 (91.4–96.3)	93.3 (90.8–95.9)	F = 2.38, *p* = 0.124
WC/HC	0.93 (0.92–0.95)	0.93 (0.91–0.94)	0.93 (0.92–0.95)	F = 0.03, *p* = 0.871
Hand-grip dynamometry, kg	45.5 (44.2–46.8)	43.6 (42.0–45.2)	41.1 (39.5–42.6) ***	F = 16.41, *p* < 0.001
TCH, mmol/L	5.7 (5.5–6.0)	5.7 (5.4–5.9)	5.8 (5.5–6.0)	F = 0.01, *p* = 0.907
HDL CH, mmol/L	0.95 (0.90–1.01)	0.99 (0.93–1.04)	1.07 (0.98–1.15) *	F = 5.72, *p* = 0.017
LDL CH, mmol/L	4.1 (3.9–4.4)	4.0 (3.8–4.3)	4.0 (3.8–4.3)	F = 0.57, *p* = 0.450
TG, mmol/L	1.4 (1.2–1.6)	1.5 (1.3–1.6)	1.5 (1.2–1.7)	F = 0.34, *p* = 0.559
Number of smoked cigarettes per day	15 (12–17)	18 (15–21)	18 (15–21)	F = 2.22, *p* = 0.138
The amount of consumed ethanol, g per week	77.3 (55.1–99.5)	119.1 (80.2–158.1)	201.0 (136.7–265.2) ***†	F = 14.59, *p* < 0.001
Physical training and sports, hours per week	2.3 (1.7–2.9)	2.5 (1.9–3.1)	1.5 (1.0–2.1)	F = 3.19, *p* = 0.075
Sedentary behavior, hours per day	6.3 (5.7–6.8)	7.8 (7.1–8.5) **	7.4 (6.7–8.1) *	F = 5.89, *p* = 0.016
Psychosocial stress score	3.2 (3.1–3.3)	2.9 (2.8–3.0) ***	2.5 (2.4–2.7) ***†††	F = 79.02, *p* < 0.001

Data are presented as M (95% confidence interval). CVD—cardiovascular diseases, SBP—systolic blood pressure, DBP—diastolic blood pressure, BMI—body mass index, WC—waist circumference, HC—hip circumference, TCH—total cholesterol, HDL CH—high density lipoprotein cholesterol, LDL CH—low density lipoprotein cholesterol, TG—triglycerides. * *p* ˂ 0.05, ** *p* ˂ 0.01, *** *p* ˂ 0.001 (versus level 1); † *p* ˂ 0.05, ††† *p* ˂ 0.001 (versus level 2). *p*-values are derived from ANOVA with post hoc comparisons using the Holm–Bonferroni method.

**Table 3:** Comparative analysis of CVD risk markers in groups of males with different vital exhaustion levels after the correction for the psychosocial stress score.

Studied Indicators	Vital Exhaustion Levels			F-Test
	1 (*n* = 97)	2 (*n* = 109)	3 (*n* = 93)	
SBP, mmHg	121 (118–124)	122 (119–124)	125 (121–128)	F = 1.58, *p* = 0.208
DBP, mmHg	81 (79–84)	82 (80–84)	84 (81–86)	F = 0.93, *p* = 0.397
Pulse, beats per minute	73 (71–75)	75 (73–77)	75 (73–77)	F = 0.86, *p* = 0.426
BMI, kg/m^2^	28.6 (27.6–29.6)	27.5 (26.6–28.4)	26.9 (25.9–28.0)	F = 2.46, *p* = 0.087
WC, cm	95.9 (93.2–98.7)	93.8 (91.3–96.3)	93.7 (90.8–96.6)	F = 0.77, *p* = 0.463
WC/HC	0.93 (0.91–0.95)	0.93 (0.91–0.94)	0.94 (0.92–0.95)	F = 0.30, *p* = 0.783
Hand-grip dynamometry, kg	45.3 (43.7–46.9)	43.6 (42.2–45.1)	41.3 (39.6–42.9) **	F = 5.36, *p* < 0.005
TCH, mmol/L	5.7 (5.5–6.0)	5.7 (5.5–5.9)	5.8 (5.5–6.0)	F = 0.10, *p* = 0.909
HDL CH, mmol/L	0.96 (0.89–1.03)	0.99 (0.93–1.05)	1.06 (0.99–1.13)	F = 1.89, *p* = 0.153
LDL CH, mmol/L	4.1 (3.9–4.4)	4.0 (3.8–4.3)	4.0 (3.8–4.3)	F = 0.22, *p* = 0.801
TG, mmol/L	1.4 (1.2–1.6)	1.5 (1.3–1.6)	1.5 (1.3–1.7)	F = 0.39, *p* = 0.680
Number of smoked cigarettes per day	15 (12–18)	18 (15–20)	18 (15–21)	F = 1.33, *p* = 0.268
The amount of consumed ethanol, g per week	70.3 (23.3–117.3)	119.5 (77.3–161.7)	209.1 (160.3–257.9) ***†	F = 7.47, *p* < 0.001
Physical training and sports, hours per week	2.5 (1.9–3.1)	2.5 (2.0–3.1)	1.0 (0.7–1.9) *††	F = 4.86, *p* = 0.008
Sedentary behavior, hours per day	6.4 (5.7–7.2)	7.8 (7.2–8.5) **	7.2 (6.5–7.9)	F = 4.23, *p* = 0.015

Data are presented as M (95% confidence interval). CVD—cardiovascular diseases, SBP—systolic blood pressure, DBP—diastolic blood pressure, BMI—body mass index, WC—waist circumference, HC—hip circumference, TCH—total cholesterol, HDL CH—high density lipoprotein cholesterol, LDL CH—low density lipoprotein cholesterol, TG—triglycerides. * *p* ˂ 0.05, ** *p* ˂ 0.01, *** *p* ˂ 0.001 (versus level 1); † *p* ˂ 0.05, †† *p* ˂ 0.001 (versus level 2). *p*-values are derived from ANOVA with post hoc comparisons using the Holm–Bonferroni method.

**Table 4:** Comparative analysis of indicators of psychosocial environment and lifestyle in groups of males with different vital exhaustion levels.

Studied Indicators	Vital Exhaustion Levels			F-Test
	1 (*n* = 97)	2 (*n* = 109)	3 (*n* = 93)	
Education	3.4 (3.2–3.5)	3.2 (3.0–3.4)	3.2 (3.0–3.4)	F = 1.74, *p* = 0.188
Social satisfaction	71.5 (68.3–74.7)	64.5 (61.2–67.9) *	51.8 (46.7–56.8) **††	F = 48.51, *p* < 0.001
The type of work	3.6 (3.5–3.8)	3.5 (3.3–3.7)	3.3 (3.1–3.5) *	F = 7.26, *p* = 0.007
Working conditions	69.8 (66.0–73.5)	61.8 (56.8–66.8) *	57.5 (52.5–62.5) ***	F = 13.40, *p* < 0.001
Working hours	8.5 (7.9–9.1)	8.7 (8.0–9.5)	8.8 (8.0–9.5)	F = 0.29, *p* = 0.588
Working relationship with managers	80.5 (77.3–83.8)	76.5 (73.0–80.0)	66.2 (61.1–71.2) **††	F = 25.95, *p* < 0.001
Working relationships with colleagues	83.9 (81.4–86.4)	80.9 (78.1–83.8)	72.7 (68.7–76.7) **††	F = 24.12, *p* < 0.001
Job satisfaction	76.8 (73.5–80.1)	68.5 (64.8–72.1) **	54.7 (49.8–59.6) **††	F = 59.08, *p* < 0.001
Lack of stress in the workplace	59.4 (55.0–63.7)	49.0 (44.5–53.4) **	46.5 (41.6–51.4) **	F = 15.45, *p* < 0.001
Spiritual needs	62.3 (58.8–65.8)	52.4 (48.3–56.5) **	46.7 (42.2–51.3) **†	F = 27.82, *p* < 0.001
Hobby	61.1 (55.7–66.4)	58.9 (54.0–63.7)	48.0 (42.5–53.6) **††	F = 11.57, *p* = 0.001
Personal happiness level	77.1 (74.2–80.1)	71.1 (68.1–74.1) **	56.9 (53.1–60.7) **††	F = 72.82, *p* < 0.001
Faith	56.8 (49.9–63.7)	57.7 (52.0–63.5)	59.2 (53.0–65.5)	F = 0.28, *p* = 0.595
Enough friends	74.9 (70.5–79.3)	70.7 (66.2–75.3)	56.7 (50.8–62.6) **††	F = 25.02, *p* < 0.001
Satisfaction with friends	76.1 (71.6–80.5)	72.2 (67.7–76.6)	61.3 (55.7–67.0) **††	F = 17.24, *p* < 0.001
Family and relatives support	90.7 (87.9–93.5)	87.7 (84.4–91.0)	81.1 (75.6–86.6) **†	F = 10.92, *p* = 0.001
Friends’ support	78.9 (74.4–83.3)	70.7 (65.9–75.5)	56.5 (50.2–62.8) ***†††	F = 34.62, *p* < 0.001
Satisfaction with the authorities	51.4 (46.4–56.4)	46.6 (42.2–51.0)	40.8 (35.4–46.2) **	F = 8.75, *p* = 0.003
Availability of essential food	82.4 (79.1–85.7)	77.2 (73.8–80.6) *	68.8 (64.3–73.4) **††	F = 24.78, *p* < 0.001
Personal safety	67.9 (64.5–71.3)	64.3 (60.8–67.8)	56.5 (52.2–60.8) **††	F = 17.49, *p* < 0.001
Living space, sq. m	75.2 (64.4–86.0)	76.9 (63.1–90.7)	56.9 (51.6–62.3) *†	F = 5.03, *p* = 0.026
Material well-being of the household	67.2 (63.9–70.6)	60.1 (56.6–63.6) **	53.5 (49.9–57.0) **††	F = 29.62, *p* < 0.001
Satisfaction with earnings	61.6 (57.1–66.1)	52.5 (47.6–57.4) *	47.2 (42.0–52.3) **	F = 16.89, *p* < 0.001
Satisfaction with housing conditions	68.7 (64.2–73.2)	65.0 (60.1–69.8)	59.0 (53.3–64.6) *	F = 7.10, *p* = 0.008
Environmental satisfaction	64.0 (59.6–68.4)	63.9 (59.9–67.8)	47.7 (42.9–52.5) **††	F = 25.65, *p* < 0.001
Satisfaction with living conditions	76.7 (73.3–80.2)	72.9 (69.3–76.5)	65.9 (61.6–70.1) ***†	F = 15.80, *p* < 0.001
Dietary diversity	74.1 (70.8–77.5)	69.2 (65.8–72.5)	59.5 (55.4–63.6) **††	F = 30.62, *p* < 0.001
Satisfaction with food intake	90.2 (87.6–92.8)	88.7 (85.9–91.5)	79.6 (75.5–83.6) **††	F = 21.07, *p* < 0.001
Intimacy issues	83.0 (79.7–86.2)	75.6 (71.4–79.9) *	64.5 (59.1–69.8) **††	F = 34.18, *p* < 0.001
Satisfaction with sleep	76.5 (72.7–80.2)	65.9 (61.2–70.7) **	53.2 (47.6–58.7) **††	F = 45.47, *p* < 0.001
Family happiness	82.3 (78.5–86.2)	76.8 (72.8–80.9)	68.6 (63.3–73.8) **†	F = 19.22, *p* < 0.001

Data are presented as M (95% confidence interval). * *p* ˂ 0.05, ** *p* ˂ 0.01, *** *p* ˂ 0.001 (versus level 1); † *p* ˂ 0.05, †† *p* ˂ 0.01, ††† *p* ˂ 0.001 (versus level 2). *p*-values are derived from ANOVA with post hoc comparisons using the Holm–Bonferroni method.

**Table 5:** Comparative analysis of indicators of psychosocial environment and lifestyle in groups of males with different vital exhaustion levels after the correction for the psychosocial stress score.

Studied Indicators	Vital Exhaustion Levels			F-Test
	1 (*n* = 97)	2 (*n* = 109)	3 (*n* = 93)	
Education	3.4 (3.2–3.6)	3.2 (3.0–3.4)	3.1 (2.9–3.4)	F = 1.29, *p* = 0.276
Social satisfaction	70.9 (66.7–75.0)	64.4 (60.7–68.2) *	52.5 (48.2–56.7) ***†††	F = 17.12, *p* < 0.001
The type of work	3.6 (3.5–3.8)	3.5 (3.3–3.7)	3.2 (3.1–3.4) *	F = 3.89, *p* = 0.021
Working conditions	68.5 (63.7–73.2)	61.8 (57.4–66.2)	59.2 (53.9–64.5) *	F = 3.38, *p* = 0.035
Working hours	8.7 (7.9–9.4)	8.8 (8.1–9.5)	8.5 (7.7–9.3)	F = 0.18, *p* = 0.837
Working relationship with managers	80.3 (76.4–84.3)	76.5 (72.7–80.3)	66.4 (62.1–70.7) ***††	F = 10.41, *p* < 0.001
Working relationships with colleagues	82.4 (79.2–85.5)	80.8 (77.9–83.7)	74.6 (71.2–78.1) **†	F = 5.39, *p* = 0.005
Job satisfaction	75.7 (71.6–79.7)	68.5 (64.7–72.2) *	56.1 (51.7–60.5) ***†††	F = 18.90, *p* < 0.001
Lack of stress in the workplace	55.5 (51.1–60.0)	48.7 (44.6–52.9)	51.3 (46.4–56.2)	F = 2.41, *p* = 0.091
Spiritual needs	62.7 (58.4–67.0)	52.6 (48.7–56.4) **	46.3 (41.8–50.7) ***†	F = 12.61, *p* < 0.001
Hobby	60.3 (54.8–65.9)	58.6 (53.6–63.6)	48.8 (43.1–54.6) *†	F = 4.27, *p* = 0.015
Personal happiness level	76.2 (72.8–79.6)	71.0 (67.9–74.1) *	58.0 (54.4–61.6) ***†††	F = 25.04, *p* < 0.001
Faith	56.1 (49.4–62.7)	57.6 (51.6–63.7)	60.1 (53.2–67.0)	F = 0.31, *p* = 0.734
Enough friends	73.7 (68.5–78.8)	71.3 (66.6–76.0)	58.2 (52.8–63.5) ***†††	F = 8.92, *p* < 0.001
Satisfaction with friends	74.7 (69.6–79.8)	72.3 (67.7–76.9)	62.9 (57.6–68.2) **†	F = 5.03, *p* = 0.007
Family and relatives support	90.1 (85.9–94.3)	87.5 (83.8–91.3)	81.8 (77.5–86.2) *	F = 3.40, *p* = 0.035
Friends’ support	77.9 (72.3–83.4)	70.8 (65.8–75.8)	57.6 (51.9–63.3) ***††	F = 11.64, *p* < 0.001
Satisfaction with the authorities	47.3 (42.3–52.3)	46.2 (41.7–50.7)	45.5 (40.3–50.6)	F = 0.12, *p* = 0.887
Availability of essential food	82.8 (78.9–86.8)	77.5 (73.9–81.1) *	68.3 (64.3–72.4) ***††	F = 11.45, *p* < 0.001
Personal safety	65.6 (61.7–69.5)	64.1 (60.6–67.6)	59.1 (55.1–63.1)	F = 2.61, *p* = 0.076
Living space, sq. m	77.3 (65.9–88.7)	77.5 (66.8–88.1)	54.4 (42.2–66.7) *†	F = 4.48, *p* = 0.012
Material well-being of the household	66.7 (63.1–70.4)	60.3 (56.9–63.6) *	54.0 (50.2–57.9) **†	F = 9.94, *p* < 0.001
Satisfaction with earnings	61.9 (56.8–67.1)	53.0 (48.4–57.6) *	46.8 (41.5–52.1) ***	F = 7.50, *p* < 0.001
Satisfaction with housing conditions	68.2 (62.9–73.5)	64.6 (59.8–69.4)	59.5 (54.1–65.0)	F = 2.25, *p* = 0.107
Environmental satisfaction	62.0 (57.4–66.6)	63.5 (59.4–67.7)	50.0 (45.2–54.8) **††	F = 9.42, *p* < 0.001
Satisfaction with living conditions	76.9 (73.0–80.8)	73.5 (70.0–77.0)	65.7 (61.6–69.7) ***††	F = 7.28, *p* < 0.001
Dietary diversity	75.0 (71.2–78.9)	69.2 (65.7–72.6) *	58.5 (54.5–62.4) ***†††	F = 16.04, *p* < 0.001
Satisfaction with food intake	90.0 (86.6–93.3)	88.7 (85.7–91.8)	79.9 (76.4–83.3) ***†††	F = 9.26, *p* < 0.001
Intimacy issues	82.7 (78.1–87.2)	76.3 (72.2–80.4) *	64.9 (60.2–69.5) ***†††	F = 13.30, *p* < 0.001
Satisfaction with sleep	74.1 (69.1–79.1)	65.6 (61.1–70.1) *	55.9 (50.8–61.1) ***†	F = 11.19, *p* < 0.001
Family happiness	80.7 (76.3–85.0)	77.0 (72.9–81.1)	70.6 (65.7–75.5) *	F = 4.04, *p* = 0.019

Data are presented as M (95% confidence interval). * *p* ˂ 0.05, ** *p* ˂ 0.01, *** *p* ˂ 0.001 (versus level 1); † *p* ˂ 0.05, †† *p* ˂ 0.01, ††† *p* ˂ 0.001 (versus level 2). *p*-values are derived from ANOVA with post hoc comparisons using the Holm–Bonferroni method.

**Table 6:** Multiple linear regression of vital exhaustion association with indicators of psychosocial environment and lifestyle in 41–44-year-old men.

Independent Variables	B (95%CI)	*p*	b	R^2^ (Partial)	R^2^(Adjusted)	VIF
Constant term	19.140 (16.546; 21.733)	<0.001	–	–	0.466	–
Personal happiness level	−0.036 (−0.056; −0.015)	0.001	−0.193	0.255		1.523
Psychosocial stress	−1.494 (−2.097; −0.891)	<0.001	−0.247	0.093		1.175
Job satisfaction	−0.027 (−0.045; −0.010)	0.002	−0.171	0.049		1.423
Satisfaction with sleep	−0.023 (−0.036; −0.009)	0.001	−0.177	0.028		1.258
Total volume of consumed ethanol, g per week	0.002 (0.001; 0.003)	0.001	0.154	0.023		1.045
Hand-grip dynamometry	−0.060 (−0.101; −0.019)	0.001	−0.138	0.016		1.045
Satisfaction with living conditions	−0.020 (−0.037; −0.003)	0.023	−0.113	0.011		1.118
Spiritual needs	−0.016 (−0.031; −0.0001)	0.049	−0.097	0.008		1.103

Dependent variable: psychosocial stress score. B—regression coefficient; CI—confidence interval; *p*-value—the probability of obtaining results at least as extreme as the observed results of a statistical hypothesis test, assuming that the null hypothesis is correct; b (beta)—standardized regression coefficient; R^2^—coefficient of multiple determination, VIF—variance inflation factor.

## Data Availability

The datasets analysed during the current study are not publicly available due to the privacy of individuals that participated in the study but are available from the corresponding author on reasonable request.
